# The Molecular Basis for Specificity at the Level of the Protein Kinase a Catalytic Subunit

**DOI:** 10.3389/fendo.2018.00538

**Published:** 2018-09-12

**Authors:** Kristoffer Søberg, Bjørn Steen Skålhegg

**Affiliations:** ^1^Department of Medical Genetics, Oslo University Hospital, Oslo, Norway; ^2^Section for Molecular Nutrition, University of Oslo, Oslo, Norway

**Keywords:** PKA, anchoring, catalytic subunit, specificity, molecular determinants

## Abstract

Assembly of multi enzyme complexes at subcellular localizations by anchoring- and scaffolding proteins represents a pivotal mechanism for achieving spatiotemporal regulation of cellular signaling after hormone receptor targeting [for review, see (1)]. In the 3′ 5′-cyclic adenosine monophosphate (cAMP) dependent protein kinase (PKA) signaling pathway it is generally accepted that specificity is secured at several levels. This includes at the first level stimulation of receptors coupled to heterotrimeric G proteins which through stimulation of adenylyl cyclase (AC) forms the second messenger cAMP. Cyclic AMP has several receptors including PKA. PKA is a tetrameric holoenzyme consisting of a regulatory (R) subunit dimer and two catalytic (C) subunits. The R subunit is the receptor for cAMP and compartmentalizes cAMP signals through binding to cell and tissue-specifically expressed A kinase anchoring proteins (AKAPs). The current dogma tells that in the presence of cAMP, PKA dissociates into an R subunit dimer and two C subunits which are free to phosphorylate relevant substrates in the cytosol and nucleus. The release of the C subunit has raised the question how specificity of the cAMP and PKA signaling pathway is maintained when the C subunit no longer is attached to the R subunit-AKAP complex. An increasing body of evidence points toward a regulatory role of the cAMP and PKA signaling pathway by targeting the C subunits to various C subunit binding proteins in the cytosol and nucleus. Moreover, recent identification of isoform specific amino acid sequences, motifs and three dimensional structures have together provided new insight into how PKA at the level of the C subunit may act in a highly isoform-specific fashion. Here we discuss recent understanding of specificity of the cAMP and PKA signaling pathway based on C subunit subcellular targeting as well as evolution of the C subunit structure that may contribute to the dynamic regulation of C subunit activity.

## The cAMP and PKA signaling pathway and cAMP receptors

**Figure 1:**
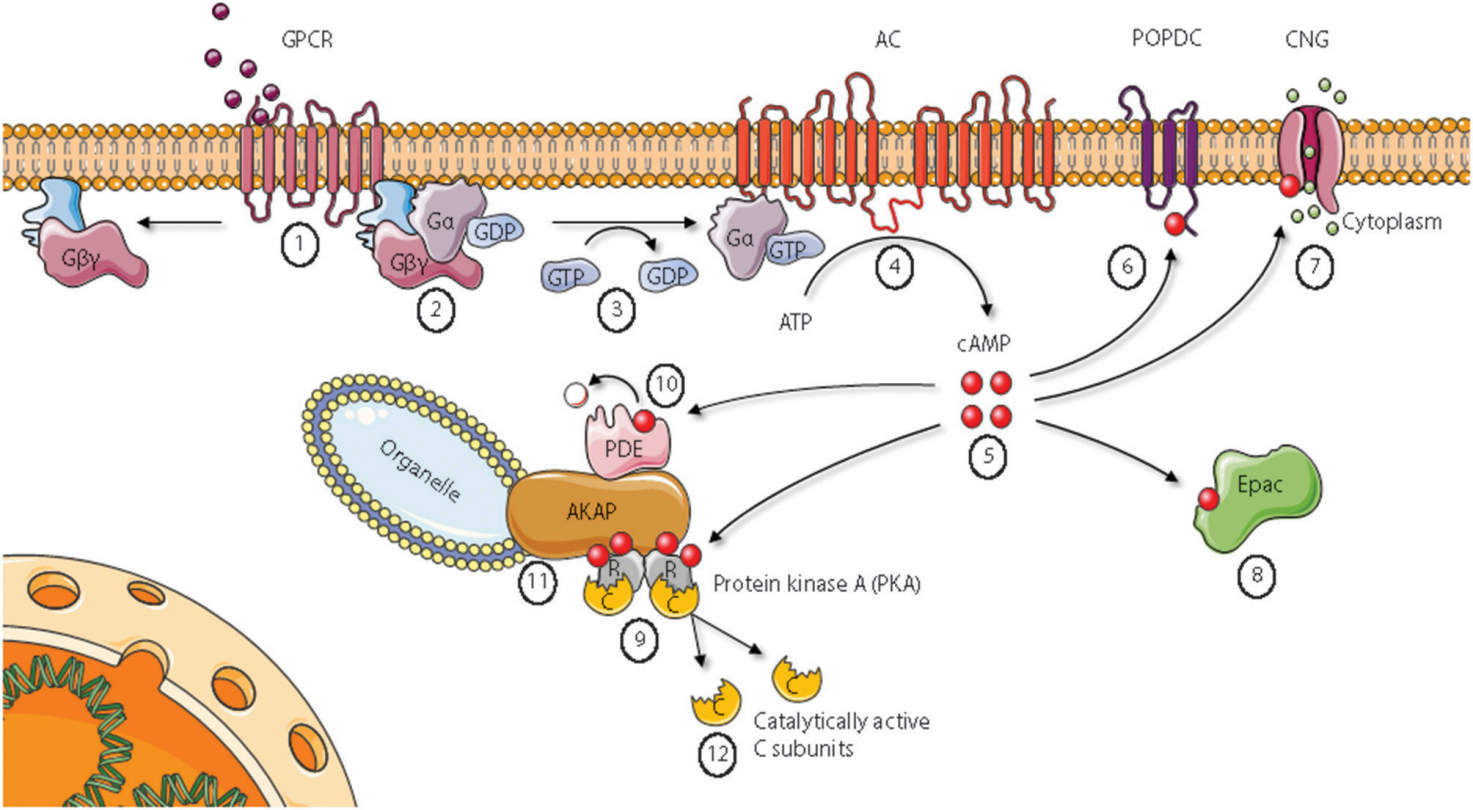
Cyclic AMP signaling pathways. Epac, Exchange protein directly activated by cAMP; AKAP, A Kinase Anchoring Protein; PDE, phosphodiesterase. See main text for details. Figure based on Wong and Scott (2). Figure created using the Servier Medical Art resource (http://www.servier.com).

In the classical conception of the Protein kinase A (PKA) signaling pathway, depicted in Figure 1, activation of PKA starts with the binding of a ligand to a seven transmembrane G protein coupled receptor [GPCR, denoted (1) in Figure 1]. Examples of such ligands include the hormones epinephrine, prostaglandin E2 (PGE2) and glucagon in addition to various neurotransmitters and other signal substances (3, 4). GPCRs have an extracellular surface recognizing ligands, and an intracellular surface interacting with membrane-bound heterotrimeric G proteins [denoted (2)]. Binding of a ligand to the extracellular part causes a conformational change in the G protein-interacting surface of the GPCR. The heterotrimeric G proteins are composed of α, β, and γ subunits. In the inactive state, all subunits are bound together, and the α subunit has a GDP-molecule attached. After activation through the ligand-bound GPCR, the α subunit exchanges its GDP-molecule with GTP [denoted (3)]. This causes dissociation of the subunits into an activated α subunit and an activated βγ complex (5, 6). There are several types of G protein α subunits (Gα), and different α subunits show distinct specificities. In the PKA signaling pathway, the stimulatory G protein known as G_S_ activates membrane (m)-bound adenylyl cyclase [AC, denoted (4)] (7).The mACs produce cAMP from ATP, and can increase the intracellular cAMP-concentration by more than twentyfold in seconds after stimulation (8). Free cAMP [denoted (5)] can bind to and stimulate several proteins, including popeye domain-containing [POPDC, denoted (6)] proteins, cyclic nucleotide-gated ion channels [CNGs, denoted (7)], exchange protein directly activated by cAMP [Epac, denoted (8)], and PKA holoenzymes [denoted (9)] (3, 4). CNGs are identified in photoreceptor cells, olfactory sensory neurons, cardiac sinoatrial node cells, kidney, liver, lymphocytes, muscle, and testis (9–13). CNG channels in visual and olfactory sensory cells convert stimuli into electrical signals through cationic influx, mainly Ca^2+^ (14). Cyclic AMP binds to Epac with high affinity and activates the Ras superfamily of small GTPases Rap1 and Rap2 (15). There are two variants of mammalian Epac, Epac1, and Epac2. Epac is involved in a range of processes, including cell adhesion, cell-cell junction, exocytosis/secretion, cell differentiation and proliferation, gene expression, apoptosis, cardiac hypertrophy, and phagocytosis (15–17). POPDC proteins are a family of membrane proteins that are found in cardiac and skeletal muscle cells, among other tissues, and are encoded by the three genes *POPDC1, POPDC2*, and *POPDC3*. They have high affinities to cAMP, and mutations in the *POPDC1* gene have been implicated in limb-girdle muscular dystrophy and cardiac arrhythmia (18, 19). Despite a vast number of reports describing these receptors for cAMP, the best studied cAMP receptor is PKA [denoted (9) in Figure 1] (3). Inactive PKA exists as a tetrameric holoenzyme of two regulatory (R) subunits in a dimer formation and two catalytic (C) subunits. The R subunits contain two cAMP binding domains (CBDs, A and B) each. Binding of cAMP to CBD B causes a conformational change of the R subunits and exposure of CBD A. The classical conception of two cAMP molecules bound to each of the R subunits is that the C subunits are released and in that way become catalytically active (20). The C subunits belong to the serine threonine protein kinase (STKs) family of enzymes, and more than 250 PKA-substrates are identified (21). The consequences of PKA activation are numerous, including regulation of metabolism, gene transcription, cell growth and division, and cell differentiation (3). Cyclic AMP binds to and is degraded by cAMP phosphodiesterases [PDEs, denoted (10) Figure 1]. The cAMP PDEs can be stimulated via phosphorylation by PKA, leading to reduced cAMP levels and consequently down-regulation of cAMP signaling in a negative feedback loop (22).

## The PKA R subunit is a major intracellular cAMP receptor

There are two major forms of the PKA holoenzyme, designated PKA type I (PKAI) and PKA type II (PKAII) (3). While PKAI is made by association of the C subunit with what is known as RI, PKAII contains RII subunits (23–25). RI and RII were initially distinguished based on their different affinity for the ion-exchange resin diethylaminoethyl (DEAE), and therefore elute at different concentrations of NaCl (26, 27). Two known isoforms of each of the RI and RII subunits are described and called RIα, RIβ, RIIα, and RIIβ, respectively (28–31). Despite that heterodimers of RIα and RIβ have been reported to form PKAI, most PKAI holoenzymes are thought to contain either RIα or RIβ homodimers (24, 32, 33). The R subunit isoforms also associate with different sets of proteins, locating the PKA holoenzymes to different subcellular compartments (34–36). All R subunits share the same overall architecture (37). The N-terminus contains a dimerization/docking domain (D/D domain). This is where the R subunits bind to each other, forming dimers. The D/D domain is also the binding site for proteins belonging to the A kinase anchoring protein [AKAP (see below for details) denoted (11)] family (38). Next to the D/D domain follows a linker region, containing a substrate/autoinhibitor site, including the primary C subunit recognition site (RS1). The RS1 site contains an actual phosphorylation site (P-site) in RII (α and β), and a pseudo site in RI. In RII the P-site is occupied by a Ser residue (38), making RII a substrate inhibitor of the C subunit. The RI subunits on the other hand represent pseudosubstrates as Ala (RIα) or Gly (RIβ). Carboxy (C)-terminally in the R subunits two cAMP binding domains are located and denoted CBD A and B, as previously described (38, 39). The various R subunits are differentially expressed in different tissues. Whereas, RIα and RIIα are ubiquitously expressed (40–42), RIβ is primarily found in brain (43), and RIIβ is expressed in endocrine tissues, brain, fat and reproductive organs (24, 44). The R subunit genes are, at least partially, non-redundant as studies of R subunit null mutated [knockout (KO)] mice reveal altered phenotypes in all four instances of R subunit ablation. The most pronounced phenotype has been described for an RIα null mutation where the mice displayed severe developmental defects and die at an early embryonic stage (45). Null mutation of the other R subunits did not show such severe phenotypes. RIIα-knockouts show reduced ocular dominance plasticity and long-term potentiation (46), as well as a predisposition to hematopoietic neoplasms (47). Ablation of RIβ was associated with reduced inflammatory response and nociceptive pain (48). In addition, RIβ KO showed a role for this subunit in tuning hippocampal synaptic plasticity (49, 50). Finally, RIIβ KO studies revealed this isoform as important in metabolic and temperature regulation (51–53) as well as motor behavior and neural gene expression (54). Interestingly, null mutation of RIIβ in mice leads to delayed cardiac aging, including resistance to age-related diastolic dysfunction and a superior global ventricular function compared to wild type (WT) (55).

Recently a report was published demonstrating that the stoichiometric relation between RI and RII is 0.6–1.5 μM revealing a 2.5-fold excess of RII in any given tissue (56). To this end, they also showed that the C subunit is greatly outnumbered by the R subunit in that the R concentration exceeds C more than 17-fold.

## PKA C subunit variants

In humans, two principal C subunit encoding genes are identified: *PRKACA* and *PRKACB*, encoding proteins Cα and Cβ, respectively. In addition, *PRKX* and the retroposons *PRKY* and *PRKACG* are identified as PKA C subunit genes. *PRKY* and *PRKACG* have not been shown to translate into proteins, whereas *PRKX* is translated into a cAMP-responsive PK which is inhibited by R subunits (57–61). At physiological conditions, *in vitro* experiments show that PRKX activity is inhibited by high affinity binding to RIα subunits, whereas RIIα subunits only show weak binding (61). Deletion of *PRKACA* in mice reveals a severe phenotype with most of the offspring dying before or at birth and during the early postnatal period (62). The mammalian *PRKACA* gene encodes two Cα variants designated Cα1 and Cα2 (63). Cα1 and Cα2 are encoded with different amino (N)-terminal ends due to the use of alternative 5′ first exons in the *PRKACA* gene. Cα1 is ubiquitously expressed, and is the main source of PKA activity in most tissues (64). Cα2 is exclusively expressed in a late stage of sperm maturation in the testis, and selective ablation of Cα2 in mice renders the mice infertile (65). Cβ exists in several splice variants due to the use of four known alternative 5′ exons, giving rise to the proteins Cβ1, Cβ2, Cβ3, and Cβ4 (66, 67). In addition, Cβ3 and Cβ4 may contain additional exons located 5′ of exon 2 termed a, b, and c (68, 69). Cβ1 is ubiquitously expressed, whereas the other Cβ splice variants are more specifically expressed in tissues like lymphoid and neuronal tissue (68, 69). Inactive Cβ subunit variants missing exon 4 have also been identified in nervous tissue (70). Genetic null-mutation of *PRKACB* results in a reduced accumulation of visceral fat in mice fed a high caloric diet, and the mice are protected from certain age-related problems, including cardiac dysfunction and hypertrophy (55).

Thus, most of the variability between Cα, Cβ, and their splice variants, is found in the N-terminal parts of these proteins. Several reports have made attempts to document functional features associated with the N-terminal end of the C subunit which is encoded by exon 1. The Cα2, Cβ2, Cβ3, and Cβ4 splice variants are lacking the N-terminal myristoylation site which is seen in both Cα1 and Cβ1 (66). C subunit myristoylation is thought to play a regulatory role in activity and localization of the C subunit (71, 72). The enzyme N-myristoyl transferase (NMT) catalyzes the covalent attachment of myristic acid onto Gly1. In the unmyristoylated form, the N-terminus of the C subunit is disordered, and it becomes ordered upon myristoylation (73). The ordered structure that is formed includes the N-terminal amphipathic helix known as the A helix (74). Experiments have shown that A kinase interacting protein (AKIP1) binds to the N-terminus of Cα1 (75), implying that the N-tail has a role in protein-protein interactions. Myristoylation leads to increased thermal stability of the C subunit, and the myristoyl group may localize to a hydrophobic pocket in the C subunit. In the myristoylated C subunit, the myristoylation site becomes solvent exposed upon binding to RII, but not RI subunits. This also leads to increased N-terminal flexibility, and the holoenzyme becomes more hydrophobic. This hydrophobicity stimulates association of PKA to membranes (76, 77). The latter has recently been substantiated in two recent publications (56, 78). In the work by Walker-Gray, they showed by inter- and intralink analysis of Cβ and RII and using XL-MS that a complex of myristoylated Cβ1 in association with RIIβ and myristoylated and acetylated (palmitate) AKAP was linked to the plasma membrane. This suggests an anchoring function of C myristoylation of the Cβ1 subunit which may also account for Cα1 which undergoes myristoylation at the same site as Cβ1.

In addition to the Gly1 myristoylation site, the two modifiable residues Asn2 and Ser10 are identified only in Cα1 and Cβ1. Asn2 may be deamidated into Asp2, whereas Ser10 is a site for autophosphorylation by PKA (71, 79, 80). Deamidated forms have been identified in about one third of all Cα1 and Cβ1 proteins isolated from hearts of pig and cattle, and the localization of the two variants were analyzed by microinjection of each fraction into the cytoplasm of NIH 3T3 cells, showing a relative lower amount of deamidated C subunit accumulating in the nucleus. This was also reflected by a lower degree of PKA-mediated phosphorylation of the transcription factor cAMP response element-binding protein (CREB) in cells microinjected with the Asp2 form (81). It is believed that deamidation of Asn2 to Asp2 occurs through the non-enzymatic β-aspartyl shift mechanism (81, 82). Ser10 has only been identified as phosophorylated in C subunit purified from *E. coli*, and has been proposed to be a transient modification in mammalian C (81). Deamidation of Asn2 seems to be a prerequisite for Ser10 phosphorylation (83). Ser10 phosphorylation is speculated to be important for C subunit membrane association controlled by a myristoyl/phosphoserine switch (74). Experiments indicate that phosphorylation of Ser10 and/or the presence of membranes may alter the conformation of the myristoyl group from “myr-in” (i.e., myristic acid bound to the hydrophobic pocket) to “myr-out” (74). Much less is known regarding the potential functions of the various N-termini of Cβ apart from Cβ1. The Cβ2 N-terminus is the longest of all the C subunits, with human *PRKACB* exon 1-2 encoding 62 residues (excluding the N-terminal Met) (69). Cβ2 has previously been identified in mammals and birds, and the N-terminus includes a stretch of residues predicted to form an amphipathic helix, possibly involved in membrane targeting (67). Human *PRKACB* exons 1-3, 1-4, a, b, and c are short exons encoding between two and eight residues. Cβ3 encodes an N-terminal Gly, but experiments indicate that this variant is not myristoylated (66). Seen together, this may suggest that features associated with the very proximal N-terminus may determine 3D structure, membrane binding capacity as well as solubility and may determine splice variant specific features.

Based on the high degree of sequence identity between Cα1 and Cβ1 it may be assumed that they have highly comparable features. However, it has been demonstrated that Cα1 has a three- to five-fold lower K_m_ for certain peptide substrates than Cβ1, in addition to a three-fold lower IC50 for inhibition by the protein kinase inhibitor (PKI) and RIIα (84).

To conclude, despite a certain degree of overlapping functions, the various isoforms constituting the PKA holoenzymes may determine the downstream effects of PKA activation (85). For example, ablation of Cα but not Cβ has been shown to lead to upregulation of the activation marker CD69 in murine lymphocytes, which was associated with increased responses to allogeneic stimulation as well as reduced sensitivity to cAMP-mediated inhibition of T cell proliferation (86). Moreover, it has recently been identified that Cβ and not Cα is necessary for inducing expression of PDE4B, a target for treatment of severe chronic obstructive pulmonary disease (COPD) (87).

## Subcellular anchoring of PKA—a means to sequester cAMP effects

A vast number of substrates and hence processes are regulated by the cAMP-PKA signaling pathway. Accordingly, a major question is, how specificity is maintained when various hormones binding to their respective receptors stimulate endogenous production of a single second messenger cAMP can elicit highly diverse signals with high precision in time and space. As understood from above, specificity is achieved at many levels, including at the level of GPCR, AC, and PDEs. Another level of specificity in the signaling pathway, which also turned out to be a general mechanism for most intracellular signaling pathways was the discovery of subcellular localization and pools of PKA differentially regulated by the cAMP-inducing receptor agonists, isoproterenol and prostaglandin E1 (PGE1) in the heart (88–91). It was later shown that the receptor for isoproterenol is located at a different place on the cardiomyocytes compared to PGE1 receptor generating distinct intracellular pools of cAMP (92, 93). In these and other studies it was also revealed that the effects of isoproterenol and PGE1 as PDE could be differentially regulated by PDE4 and PDE3, respectively (94). The differential effects of agonists and PDE3 strongly points to compartmentalization of cAMP, but also to the colocalization of enzyme complexes, such as PKA and PDEs. Later it has been demonstrated that this occurs through subcellular targeting of PKA, PDEs and other proteins important to signal transduction to a large group of proteins which are designated AKAPs (A kinase anchoring proteins) (95–97). AKAPs are a group of structurally diverse proteins, with a common function, targeting of the R subunit and hence, confining PKA to discrete locations within the cell. R subunit binding is conveyed by a targeting domain that anchors the R subunit, and thereby PKA to specific subcellular locations. More than 50 AKAPs have been identified that are expressed in virtually all tissue and cell types examined. These complexes are typically associated with of PKAII holoenzymes as AKAPs were initially shown to bind to the RII subunit with an up to several hundred fold higher affinity compared to the RI subunit (98). The nature of the RII binding domain was initially revealed by helical wheel analysis of AKAP13 (also known as AKAP-Lbc) and a peptide termed human thyroid 31 (Ht31) which was derived from the predicted RII binding domain of AKAP13. It is however well established that the PKAI holoenzymes, which are mainly identified in the soluble fraction of the cell, may bind to RI- and dual-specific AKAPs (95, 96). They can localize to most cellular organelles including the plasma membrane (AKAP7 and AKAP5), the cytoskeleton (AKAP12 and AKAP13), mitochondria (AKAP1), the Golgi apparatus (AKAP9), vesicles (AKAP11) as well as the nucleus (AKAP 8) (99). Because AKAPs bind RII and RI with different affinities, AKAPs are normally subdivided into three classes; RI-, RII-, or dual-specific (100). The RII binding domain consists of an amphipathic α-helix of 14–18 amino acids that binds to the four-helix bundle formed by the D/D domains of the R dimers. AKAPs may also bind to the D/D domain of RI. However, it has been shown that several dual-specific AKAPs have an additional PKA binding determinant termed RI specifier region. It is located outside the common amphipathic helix motif and contains basic residues that associate specifically with RI and not RII (100). AKAPs also serve as signaling nodes for several participants in the cAMP signaling cascade, including GPCRs (101), ACs (102), Epac (103), protein phosphatatses (PPs) (104), and PDEs (105). In this way AKAPs function as scaffolds that orchestrate signaling events in space and time by forming multicomponent complexes. Thus, AKAPs facilitate cross talk and integration of different signaling pathways and are often referred to as signalosomes (99). This function is well documented by the dynamic association of RIα in the holoenzyme form, associated with C, and in non-C bound form and AKAP11 (106). Day and coworkers demonstrate an AKAP-dependent localization of RIα to multivesicular bodies (MVBs). Binding of RIα to AKAP11 occurs both in RIα holoenzymes and in RIα dimers not bound to C subunits. However, recruitment to MVBs takes place exclusively after the C subunits are released. Association with MVBs is reversed when C subunits reassociate with the RIα subunits. This documents that AKAPs may direct R subunit (in this case RIα) functionality after C subunit dissociation.

## C kinase anchoring proteins—C-KAPs

**Figure 2:**
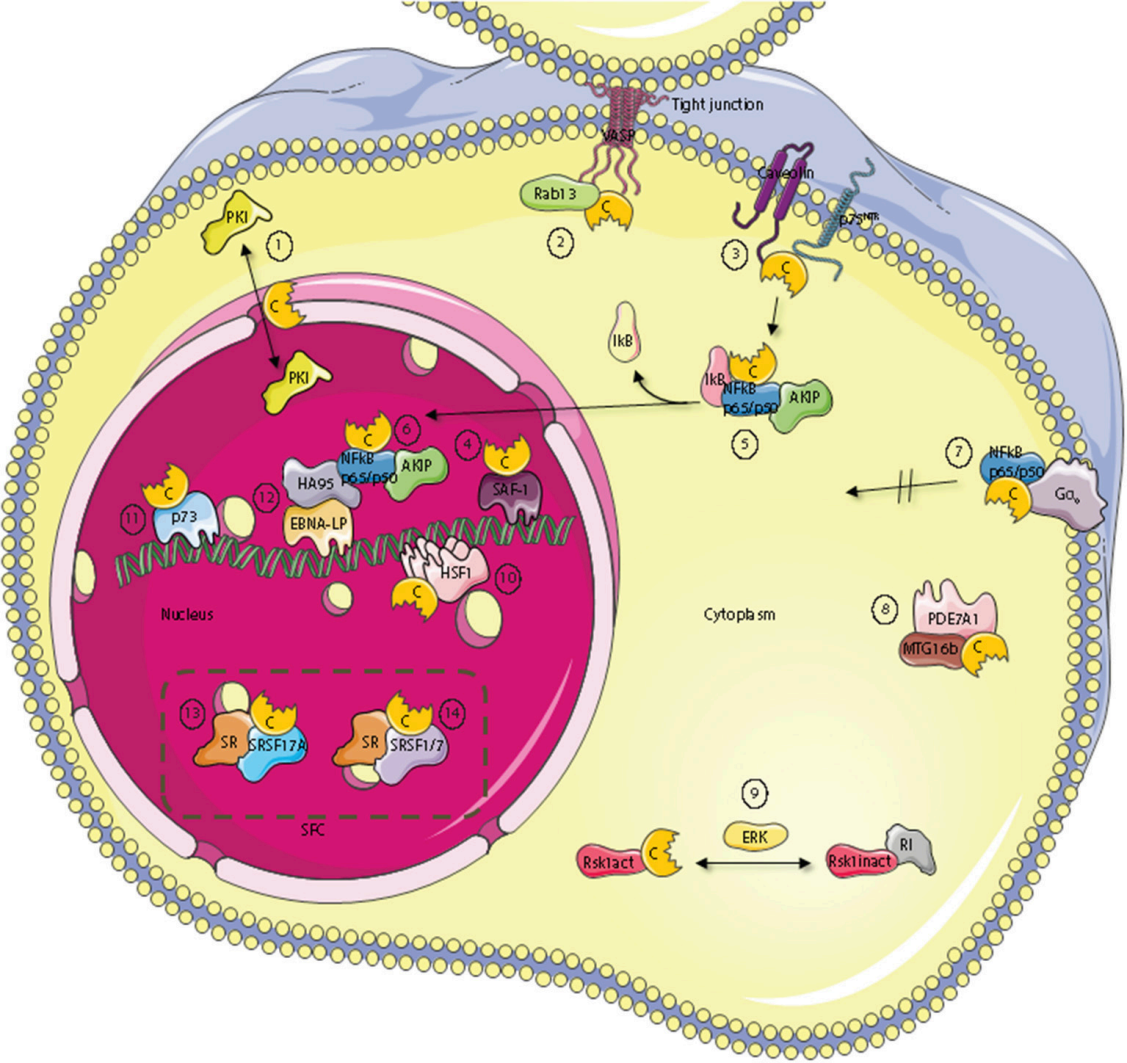
Subcellular localization of C kinase anchoring proteins—C-KAPs. The PKA C subunit associates and regulates the activity of proteins located to multiple cellular compartments and molecules. These compartments include the outer cell membrane, the cell cytoplasm and the cell nucleus. In the nucleus, C-KAPs co-locate the PKA C subunit with DNA and components of the splicing factor compartment (SFC). In the cytoplasm the PKA C subunit in addition to associate with the PKA R subunit interacts with the PKA inhibitor PKI (1), the small G protein Rab13 (2), PDE7A1 (8) the Rsk1 kinase through regulation by ERK (9), and finally IkB (5) which is a component of the cytoplasmic NFkB/AKIP complex. In the outer membrane compartment PKA C subunits associate with caveolin-1, p75^NTR^ (3), and the heterotrimeric G protein Gα0 (7). In the nucleus, the PKA C subunit regulates DNA activity through interaction with SAF-1 (4), HSF-1 (10), HA95 (12), and p73 (11). Finally, the PKA C subunit is also involved in regulating mRNA splicing in SFC by direct interaction with serine and arginine (SR) proteins such as SFSR17A (13), SRSF1 and SRSF7 (14).

Proteins that bind the PKA C subunits are diverse and located to all parts of the cell, e.g., the outer membrane, the cytoplasm, and the nucleus. For this group of proteins, we propose the term C kinase anchoring proteins, C-KAPs. C-KAPs are all proteins that bind directly to the C subunit and affect its localization. They include substrates and pseudosubstrates such as the RII- and RI subunits, respectively, as well as proteins binding to other parts of the C subunit outside of the active site cleft, such as AKIP. This means we define C-KAPs as distinct from proteins targeting the R subunits to subcellular compartments through AKAP interactions and proteins that interact transiently as substrates for the C subunits without targeting the C subunit to subcellular structures or other scaffolds. Given this definition, the first C-KAP apart from the R subunits to be described was PKI [Figure 2 (1)] (107). There are three isoforms of PKI; PKIα, PKIβ, and PKIγ, and all act as physiological inhibitors of PKA by serving as pseudosubstrates for the C subunit (108, 109). As all PKA C subunits except Cγ are identical in the catalytic cleft it is assumed that PKI binds with the same affinity and specificity to all Cα and Cβ isoforms, a feature which is confirmed for Cα1 and Cα2 (110). With respect to the Cγ subunit, it is not inhibited by PKI (111). The PKI isoforms contain a nuclear export signal (NES) and is important in active transport of free C subunits from the nucleus (112). No apparent phenotypical effects of ablating the different PKI isoforms in mice have been demonstrated, even though their different expression patterns imply specific roles (113, 114).

Several C-KAPs are expressed in the cytoplasm. One such protein is the small GTPase Rab13 which is involved in tight junction dynamics. Vasodilator-stimulated phosphoprotein (VASP) is a PKA substrate and a key actin cytoskeletal remodeling protein (115). GTP-bound Rab13 interacts directly with PKA Cα and inhibits PKA dependent phosphorylation and tight junction associated VASP. This leads to functionally defect tight junctions (116) [Figure 2 (2)].

A number of components of the cAMP signaling cascade have been localized to caveolae which are a part of lipid rafts and function as endocytic and exocytic compartments at the plasma membrane of most cells. Caveolin-1, which is the main component of caveolae may be considered a C-KAP, as it binds and targets the C subunit to the cell membrane (117, 118). The PKA C subunit associates through binding of the Caveolin-1 scaffolding domain on the C-terminus and the binding has been shown to inhibit C subunit enzymatic activity (118) [Figure 2 (3)]. This may explain why Caveolin-1 KO mice revealed hyperactive PKA (119). Caveolin-1 KO mice also have abnormal lipid homeostasis with phenotypic characteristics associated with type II diabetes, a feature that has been linked to disturbances in PKA-mediated phosphorylation. To this end it has been shown that Caveolin-1 bound C subunit also binds to perilipin, which is a protein that covers and protects lipid droplets, can be phosphorylated by PKA, and co-immunoprecipitates with Caveolin-1-PKA C from adipocytes upon β-adrenergic stimulation (120). Moreover, it has been shown that PKA-mediated perilipin phosphorylation and subsequent release of lipids from lipid droplets is dependent on Caveolin-1 induced formation between PKA C and perilipin (119). Furthermore, optic atrophy 1 (OPA1), a protein known to regulate mitochondrial dynamics, was identified as a dual-specificity AKAP that associates with lipid droplets. OPA1 targets PKA to lipid droplets for the purpose of hormonal control of perilipin phosphorylation and lipolysis (121).

p75 neurotrophin receptor (p75^NTR^) may be considered a cell membrane-bound C-KAP [Figure 2 (3)]. Ligands for p75^NTR^ are the neurotrophins, a class of growth factors that regulate the survival, differentiation, growth, and apoptosis of neurons. p75^NTR^ has been shown to interact with and be phosphorylated by the PKA Cβ4ab splice variant (122). Moreover, activation of the cAMP and PKA pathway was required for translocation of p75^NTR^ to lipid rafts and for the receptors biochemical and biological activities. Furthermore, p75^NTR^ has also been demonstrated to associate with Caveolin (123).

An important downstream effect of neurotrophin bound to p75^NTR^ is to activate the nuclear factor κ-light-chain-enhancer of activated B cells (NFκB) pathway (124). NFκB has been implicated as an important transcription factor for serum amyloid A (SAA) which is a family of plasma proteins that is linked with several inflammatory diseases, including amyloidosis, rheumatoid arthritis and atherosclerosis. Activation of has further been linked to SAA-activating factor 1 (SAF) which has been shown to associate with PKA Cα (125) [Figure 2 (4)]. In line with this, activation of SAF-1 is cAMP-dependent and cAMP increases the transactivating and DNA-binding properties of SAF-1 (126).

NFκB is a member of the Rel family of rapid-acting transcription factors. The NFκB p65/p50 dimer is kept inactive in the cytosol in complex with the protein inhibitor of κB (IκB). IκB which masks the nuclear localization signal (NLS) of NFκB interacts with Cα1 when IκB is associated with NFκB. Cα1 is inactive until stimulation and activation of NFκB by agents such as cytokines and reactive oxygen species (ROS). This leads to the release and degradation of IκB with subsequent cAMP-independent activation of PKA C [Figure 2 (5)]. The C subunits have been shown to phosphorylate Ser276 in the p65 subunit. This enhances NFκB transcriptional activity markedly and increases the expression of NFκB downstream target genes involved in e.g. inflammation, cell proliferation and survival. Interestingly, AKIP which was first identified as a PKA C-binding protein (75) is also a p65 interaction partner (127) [Figure 2 (5)]. According to this observation it has been suggested that binding of the C subunit and AKIP to the p65 subunit encompass PKA-dependent phosphorylation and regulation of NFκB dependent transcription (127). Together this also suggests that AKIP1 acts as a molecular scaffold that simultaneously binds and coordinates PKA C and p65 activity. In line with this it was shown that AKIP1 regulates NFκB nuclear translocation (128) [Figure 2 (6)]. Another aspect of PKA C and NFκB interaction has been documented according to the fact that this complex is activated when the heterotrimeric G protein Go interacts directly with the C subunit independent of the R subunit [Figure 2 (7)]. This suggests that Gαo is a C-KAP as well. Interestingly, when C binds to Gαo this prevents C from translocating to the nucleus but does not inhibit catalytic activity (129). The fact that Gαo interacts with both PKA holoenzyme and free C subunits provides evidence that active C can be released from R and positioned in an active state in close proximity to relevant substrates as well as the R subunit for rapid association, holoenzyme formation, and presumably inactivation when cAMP is degraded. With respect to cAMP degradation, the PDE variant PDE7A1 does not only degrade cAMP to regulate PKA activity (130). PDE7A1 also regulates C subunit activity through direct interaction between the C subunit and the N-terminus of PDE7A1 [Figure 2 (8)]. The PDE7A1 N-terminus which contains two PKA pseudosubstrate sequences may be considered a C-KAP (131). It should also be mentioned that PDE7A1 interacts with myeloid translocation gene 16b (MTG16b) which is a dual specific AKAP (132, 133). Although the function of the AKAP-MTG16b/PDE7A1/PKA complex is not fully understood [Figure 2 (8)], it is likely that this structure as is the case with the Gαo/NF-κB/C-subunit complex discretely regulates spatiotemporal effects of PKA. Furthermore, as the Gαo/NF-κB/C-subunit complex is also identified in T cells it is suggested that it may be a key factor of the signaling complex regulating T cell activation (134).

A major signaling pathway in eukaryotic cells is the mitogen-activated protein kinase (MAPK) pathway which is implicated in regulating cell growth, differentiation and apoptosis. A typical MAPK pathway consists of a cascade of three successive phosphorylations exerted by a MAPK kinase kinase (MAPKKK e.g., Raf), a MAPK kinase (MAPKK, e.g., Mek), and a MAPK (e.g., Erk). MAPKs target other proteins such as kinases known as the MAPK-activated protein kinases (MAPKAPK) that belong to the Ca^2+/^calmodulin-dependent protein kinases. Among these kinases we find the ribosomal-S6-kinases (RSKs) which represent points of cross-talk between the PKA pathway and the MAPK pathway. RSK1 is a serine/threonine kinase with important functions in cellular growth control and proliferation (135). RSK1 interacts with PKA RI in its unphosphorylated and inactive state while activated RSK1 binds directly to C subunits (136) [Figure 2 (9)]. Binding of RSK1 to RI decreases the interaction between RI and PKA C subunit while the binding of active RSK1 to the C subunit increases the interaction between the PKA C and R subunits. These findings indicate that RSK1 acts both as a C-KAP and AKAP involved in a negative feedback loop of PKA activity where PKA can trigger phosphorylation of RSK1 through activation of ERK. Phospho-RSK1 will then promote association of the PKA holoenzyme (137). At the same time, through the association with PKA, D-AKAP1 serves as a necessary nuclear anchor for RSK1. D-AKAP1 also binds protein phosphatase 2A (PP2A) that can dephosphorylate and thus regulate RSK1 activity (138).

In response to heat shock or other physiological stresses, heat shock factor (HSF) rapidly oligomerises into DNA-binding trimers, accumulates in the nucleus and acts as a transcription factor that regulates the expression of heat shock proteins (HSP). HSPs belong to a class of functionally related proteins involved in the folding and unfolding of other proteins (139). This process involves activating posttranslational modifications and the HSF isoform HSF1 has been shown to be sumoylated and acetylated as well as heavily phosphorylated (140). Hyperphosphorylation of HSF1 is correlated with transcriptional competence and slow dissociation of active trimers (141). Moreover, later it has been shown that it acts as a C-KAP as HSF1 binds to PKA Cα and is activated by PKA-dependent phosphorylation [Figure 2 (10)]. This again permits HSF1 to accumulate in the nucleus where it activates transcription of HSP70.1 (142). The latter is consistent with the observation that the cAMP and PKA-pathway regulates HSP70 promoter activity (143).

Another protein important for the regulation of cell growth and proliferation is p73, which is a structural and functional homolog of the widely known p53 tumor suppressor protein (144). Evidence suggests that p73 can bind to the p53-responsive element and transactivate an overlapping set of p53 target genes, thus, leading to the induction of G1/S cell cycle arrest. p73 is expressed as multiple isoforms arising from alternative splicing of the primary p73 transcript (p73α, p73β, p73γ, p73δ, p73ε, p73η, and p73ζ). p73α, but not p53, has been demonstrated to be phosphorylated by and bind to the PKA Cβ subunit, and may therefore be considered a C-KAP [Figure 2 (11)] (145).

Taken together these reports demonstrate regulation of activity and location of C and its interaction partners both in the cytoplasm as well as the nucleus. It has further been reported that the PKA holoenzyme may localize within the nucleus (146). Nuclear localization of the PKA holoenzyme is however controversial and remarkably few AKAPs have been demonstrated to reside within this cellular compartment in interphase cells. The present dogma suggests that PKA acts in the nucleus when the C subunit is transferred to the nucleus upon cAMP stimulation. Hence, it is likely that several nuclear C-KAPs exist. Homologous to AKAP95 (HA95), which shows high homology to AKAP95, binds the PKA C subunit independent of R in the nucleus and may be considered a C-KAP (147) [Figure 2 (12)]. HA95 is a nuclear protein and contains a NLS. The gene encoding HA95 probably arose due to a gene duplication of AKAP95 and may have survived evolution because it solely binds the C subunit and does not compete with AKAP95 for R subunit binding (147). HA95 activity is associated with several nuclear processes, including shuttle protein activity by binding to RNA helicase A and the activation of the retroviral constitutive transport element (148). HA95 is also involved in regulation of nuclear envelope dynamics (149), DNA replication (150), and regulation of the histone deacetylase (HDAC) pathway (151). Although conclusive evidence lacks, these reports may imply that there is a direct link between cAMP and PKA-dependent regulation of various nuclear events and C subunit binding to HA95. However, support for a direct binding of C to HA95 comes from experiments on Epstein-Barr virus (EBV) infected B cells. We have demonstrated that binding of the C subunit to HA95 is required for EBV infection (152, 153). In these experiments, HA95 was also shown to co-immunoprecipitate with the EBV co-activating nuclear protein EBNA-LP from EBV-transformed lymphoblastoid cells. It has been suggested that HA95 binds to the EBNA-LP repeat domain which is the principal co-activator of virus transcription. In this way, EBNA-LP co-localizes with HA95 and the C subunit [Figure 2 (12)]. Moreover, C subunit expression down-regulated the strong co-activating effects of EBNA-LP with EBNA-2 which also regulates the expression of the EBV oncogene, LMP1. Interestingly, over-expression of the C subunit or HA95 down-regulates LMP1 expression in EBV-infected cells. Together this demonstrates a functional consequence of co-locating C with C-KAP, in this case with HA95 and EBNA-LP to affect transcription from specific promoters. In this context it is interesting to note that DNA-protein kinase (DNA-PK) also associates with the EBNA-LP/HA95/PKA-C subunit complex (154). Together this demonstrates that PKA targeting is involved in regulating the complex processes of gene transcription. An interesting question is then, whether PKA targeting is required for precursor-messenger RNA (pre-mRNA) transport and processing.

During the last decade accumulating evidence shows that PKA C interaction with other proteins is involved in regulating precursor-messenger RNA (pre-mRNA) splicing. Pre-mRNA splicing is a highly complex process in the eukaryotic cell and involves a tight regulation of protein-protein, protein-DNA and protein-RNA interactions in time and space (155). We have shown that PKA C co-locates with splicing factor compartments (SFC) in the nucleus and that PKA promotes distal splicing of the E1A minigene. We were also able to show that some of the most heavily PKA phosphorylated proteins belong to the family of serine and arginine rich proteins, or SR-proteins. Some of the proteins phosphorylated were the SR splicing factors SRSF1, SRSF2, and SRSF9 (156). It has previously been demonstrated that the SR proteins have central roles in the regulation of constitutive and alternative RNA splicing (157). Later it has been demonstrated that both R and C subunits of PKA can bind to splicing factor arginine/serine-rich 17A (SFSR17A) defining this protein as an AKAP as well (158) [Figure 2 (13)]. SFSR17A targets PKA in close proximity to several members of the SR family of proteins. Furthermore, several independent reports show that the PKA C subunit interacts with the SR protein SRSF1 (159–161). PKA-dependent phosphorylation of SRSF1 was found to enhance its RNA-binding capacity (159), and to modulate its activity as a splicing regulator (159–161). Furthermore, another SR protein, SRSF7 is not only phosphorylated by but also interacts with Cα1 (161) [Figure 2 (14)]. Also, the adenoviral splicing factor L4-33K is phosphorylated by PKA and DNA-PK. Interestingly, the two kinases have opposite effects on alternative splicing of virus-specific proteins (154). Finally, the G-patch domain and KOW-motifs containing protein (GPKOW) has been shown to be a nuclear protein that binds RNA in a PKA-regulated fashion (162). For a summary of known C-KAPs see Table 1.

**Table 1:** Overview of selected C kinase anchoring proteins (C-KAPs).

**Protein**	**Primary functions**	**Localization**	**Site of interaction**	**Effects of interaction**	**References**
Protein kinase inhibitor peptide (PKI)	Inhibits PKA catalytic activity	Cytoplasm and nucleus	Pseudosubstrate sequence of PKI interacts with the substrate binding sites in the catalytic cleft of the C subunit	Inhibits PKA C phosphorylation PKI contains a NES that assists export of the C subunit out of the nucleus	(107–109, 112–114)
Ribosomal S6 kinase1 (RSK1)	Ser/Thre kinase that phosphorylates some of the same targets as PKA	Cytoplasm and nucleus	Cα1 binds to the 13 C-terminal amino acids in Ser732 phosphorylated RSK1	Inhibits C subunit activity by stimulating the reassociation of the R and C subunits	(135–138)
Heat shock factor 1 (HSF1)	Controls heat shock responses Implicated in cancer and neurodegenerative disease	Cytoplasm and nucleus	Not known	Phosporylation by PKA on Ser320 in HSF1 is important for nuclear localization of HSF1 Unknown effects on the C subunit	(139–143)
A-kinase interacting protein (AKIP)	Interacts with the p65 subunit of NF-κB Brings the C subunit in close proximity to NFκB for phosphorylation by the C subunit	Nucleus	The C-terminal end of AKIP interacts with the N-terminal α-helix (aa 14–39) of the C subunit	Targets and retains Cα1 in the nucleus	(75, 127, 128)
Inhibitor of NF-κB (IκB)	Inhibits NF-κB-dependent transcription by binding to its DNA-binding site	Cytoplasm	Unknown part of IκB binds to the N-terminal end of the C subunit (aa 46–76)	Inhibits C subunit activity by blocking the ATP-binding site cAMP independent mechanism	(127)
Heterotrimeric G protein Gα_O_	Heterotrimeric G protein hydrolysing GTP to GDP. Links GPCR to enzymes such as AC and phospholipases.	Cytoplasm	The GTPase domain in Gα_O_ interacts with an unknown part of Cα1	Targets active Cα1 to the cytoplasm	(129)
Phoshodiesterase 7A1 (PDE7A1)	Hydrolysis of cAMP to 5′ AMP Targets the C subunit to AKAP CBFA2T3, independent of the R subunit.	Cytoplasm	Two N-terminal pseudosubstrate sequences in PDE7A1 interacts with the C subunit	Inhibits C subunit activity by blocking the substrate binding site	(130–133)
Caveolin-1	Principal components of caveolae membranes and involved in receptor-independent endocytosis Forms a complex with the C subunit to promote phosphorylation of Perilipin	Cytoplasm	Scaffolding and C-terminal domain of Caveolin-1 binds to unknown part of the C subunit	Inhibits C subunit activity	(117–120)
Homologous to AKAP95 (HA95)	Regulation of DNA replication, nuclear envelope dynamics and HDAC pathway.	Nucleus	Not known	Targets the C subunit to the nucleus	(147–154)
Rab13	GTPase with regulatory function in epithelial tight junctions	Cytoplasm	Not known	Inhibits C subunit-dependent phosphorylation of VASP in epithelial cells	(115, 116)
p73	Transcription factor that promotes apoptosis and cell cycle arrest	Nucleus	N-terminal (aa 63–130) and C-terminal (aa 469–636) interact with unknown domain of the Cβ1 subunit	C subunit-dependent phoshorylation inhibits transcriptional and proapoptotic activity of p73	(144, 145)
SRSF7	Several functions in RNA processing, including pre-mRNA splicing	Cytoplasm and nucleus	Not known	Upregulated PKA activity prevents inhibition of tau exon 10 inclusion by SRSF7	(161)
GPKOW	RNA-and protein-binding protein	Nucleus	Not known	C-subunit-dependent phosphorylation of Ser27 and Thr316 inhibit GPKOWs ability to bind total RNA	(162)
SRSF1	Several functions in RNA processing, including pre-mRNA splicing	Cytoplasm and nucleus	Not known	C-subunit-dependent phosphorylation of SRSF1 modulates its effect on splicing	(156, 157)

## Determinants for the regulation of C subunit activity

Together, these reports imply that PKA is involved in the regulation of multiple steps in the splicing process by phosphorylation. This also demonstrates that the process requires discrete positioning of the C subunit in proximity to relevant substrates. This raises the question, what are the determinants in the C subunit dictating localization and binding to other proteins? The inhibitory site of the R subunit vs. the C subunit is a distinguishing feature between the RI and RII isoforms (163). The RII subunits have a phosphorylation site (P-site) in their inhibitor motif and therefore act both as substrates and inhibitors. By contrast, the RI subunits are encoded with Ala or Gly at the RII P-site and thus, act as pseudosubstrates. Despite that both RI and RII bind C with a sub nanomolar Kd (0.1 nM) the mechanism of binding is different. RI, but not the RII subunits, requires ATP and divalent cations to form stable interactions (31, 164).

Moreover, autophosphorylation of the P-site in RII subunits promotes holoenzyme dissociation.

The structure of the C:RI, C:RII and C:PKI interactions show major similarities in how catalytic activity is regulated as the regions P−3 to P+1 in the C subunit are occupied by both RI, RII as well as PKI upon binding. It should also be noted that the mechanism of PKI binding to the active cleft in C is more similar to that of RI than RII, both being pseudosubstrates and needing ATP and two divalent cations for stable interactions.

As several of the C-KAPs apart from R and PKI also inhibit the C subunit upon binding, a highly relevant question is whether C-KAPs share the same mechanism of inhibition as R and PKI. In the case of Caveolin-1, it has been demonstrated that residues between Trp85 and Trp98 associate with several amino acids scattered between Phe54 and Tyr247 in the C subunit (165). Moreover, when PDK1 phosphorylates the C subunit at Thr197, this involves anchoring of the C-terminal hydrophobic motif Phe-x-x-Phe to the small lobe of PDK1 (166). It has also been shown that Goα through its GTPase domain interacts directly with Cα1 (129). However, few of these papers mapped the domains in the C subunit responsible interaction with the C-binding protein. One example of mapping of such domains has been done by studying the Cα1:AKIP1 interaction (75, 167). In these two reports it was demonstrated that AKIP1 binds to the A helix residues 15–30 in the Cα1 subunit and that this interaction is the mechanism responsible for targeting the C subunit to the nucleus. This may suggest that the C subunit N-terminal end is involved in targeting the kinase to relevant target proteins and hence subcellular compartments independent of the R subunit. However, the specificity in this form of targeting remains elusive. It has been speculated that splice variant-specific features of the C subunits which are associated with the first exon may play an important role. In addition, anchoring of Cα1 and Cβ1 may involve myristoylation (56).

## Catalytic subunit structural features as determinant for PKA specificity

**Figure 3:**
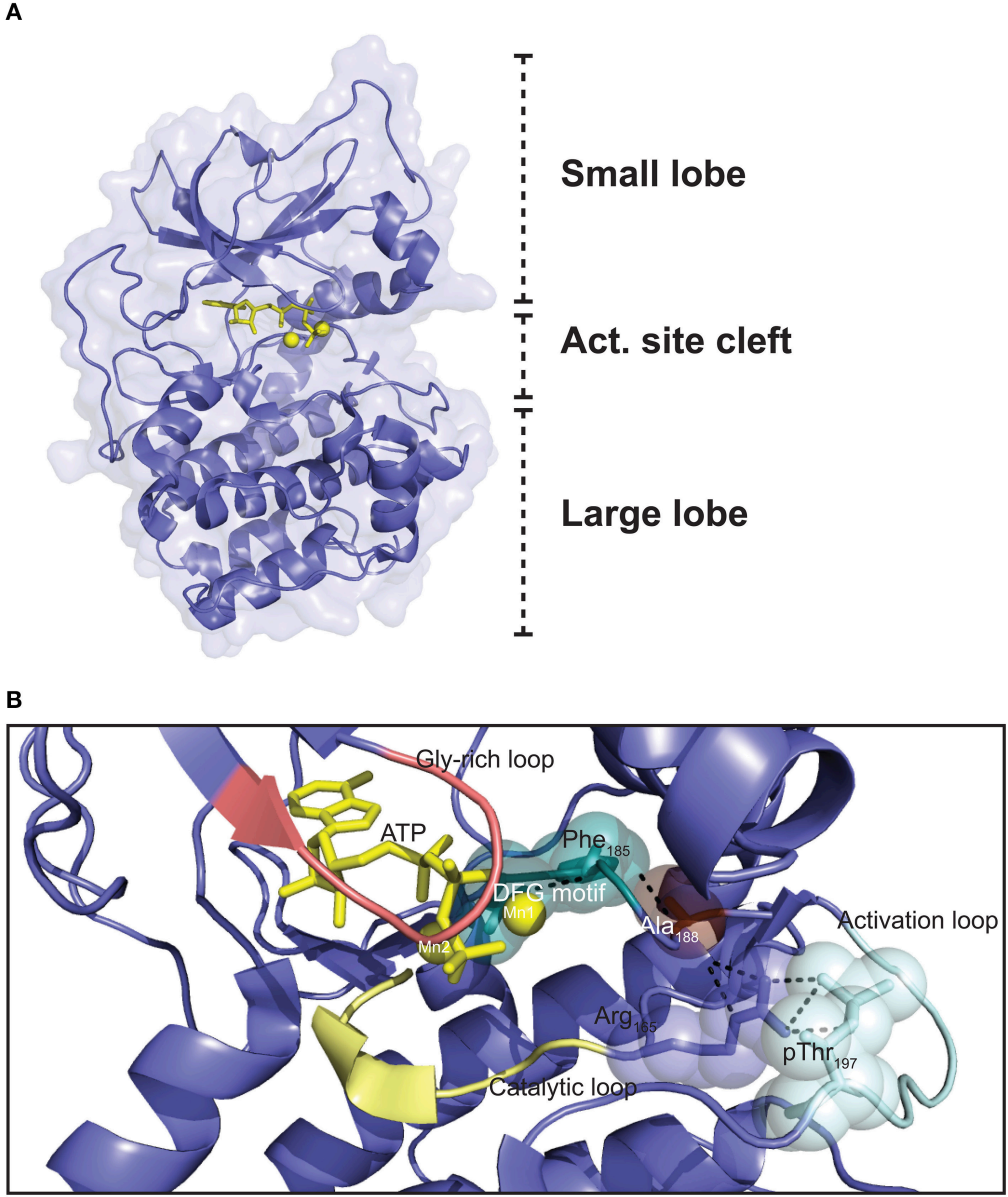
Three dimensional structure of the PKA C subunit. **(A)** The C subunit is composed of a small lobe, large lobe, and an active site cleft with a binding site for an ATP molecule (yellow sticks) and two Mg2+ ions (yellow spheres). The figure is based on the experimental structure with Protein Data Bank (PDB) identifier 3FJQ (169). **(B)** Schematic representation of the active site cleft of PKA Cα1. Motifs and residues described in the text are indicated. Dashed lines indicate the chain of interactions leading from pThr197 to Phe185 in the DFG motif when the enzyme is in the active conformation. The structure is solved with Mn^2+^ as the divalent cations, although Mg^2+^ is thought to be the most relevant biological chelating agent (170). ATP and the Mn^2+^ ions are shown in yellow, and the DFG motif (teal), Gly-rich loop (salmon), catalytic loop (yellow), and activation loop (cyan) are also highlighted. PDB identifier 3FJQ (169).

The Cα1 and Cβ1 proteins are both made up of 350 aa residues, containing only short N- and C-terminal segments that are not part of a conserved catalytic core comprising Cα1/Cβ1 residues 40–300 (168). Whereas, the flanking N- and C-terminal sequences are more specific to the C subunit, and hence vary considerably the catalytic core consists of a large C-terminal lobe which is also named the C-lobe or large lobe, and a smaller N-terminal lobe also known as the N-lobe or small lobe (Figure 3A) (171). The large lobe is shaped by seven α-helices in addition to contributing to the formation of a sheet of four antiparallel β-strands in the surface facing the cleft between the two lobes (172). The large lobe contains most of the substrate-binding surface as well as much of the catalytic machinery (85). The small lobe, mostly composed of β-strands, five in total in an antiparallel orientation in a single sheet, contains most of the residues binding to ATP (172). The small lobe also contains two helices; the αB and αC helix (172). The segment that connects the small and large lobes is called the hinge region (173). The cleft between the small and large lobes has been termed the active site cleft. This cleft shapes the ATP binding pocket, with the γ-phosphate facing outwards toward the solute and substrate-binding site. Most of the highly conserved residues of Cα and Cβ are located around the active site cleft. ATP functions as “glue” coupling the small and large lobe together, partially through forming hydrogen bonds with residues in the hinge region (173). Hence, in the absence of ATP the catalytic cleft is relatively uncoupled (174).

Several structural motifs and residues important for function are identified in the catalytic core (Figure 3B). The activation loop (residues 191–197) includes Thr197, which has to be phosphorylated in order for the C subunit to be catalytically active. Phosphorylation of Thr197 leads to a cascade of interactions in the protein structure, contributing to organizing the kinase in the active conformation (see below) (175). The catalytic loop (residues 166–171) contains several residues important for catalysis. One of the most flexible segments of the catalytic core is the glycine-rich loop (residues 50–55) (172, 176). This loop is important for positioning of the γ-phosphate of ATP for phosphoryl transfer during catalysis (174). The Mg^2+^ positioning loop (residues 184–187), containing the DFG (Asp-Phe-Gly) motif (residues 184–186), is among the most conserved segments encompassed in the catalytic core. The catalytic core not only binds an ATP molecule, but also has binding sites for two divalent cations, preferentially magnesium ions (Mg^2+^) termed activating and inhibitory Mg^2+^, or Mg1 and Mg2, respectively, which are necessary for catalysis (170), but also RI binding (177). The traditional view has been that in presence of ATP, the binding affinity for Mg1 is higher than for Mg2, and binding of Mg2 is thought to stabilize the protein but also to inhibit catalysis (170, 178–180). Other reports have suggested that the presence of two Mg^2+^ in the active site of other related eukaryotic protein kinases (ePKs) such as CDK2 is necessary for efficient phosphoryl transfer, but also leads to stabilizing the binding of ADP to the active site, which is the rate-limiting step of the catalytic cycle (181). Moreover, Ca^2+^ may exist in local high concentrations near calcium channels, and it is proposed that fluctuations in Ca^2+^-concentrations may influence C subunit function through reduced enzyme activity and PKI affinity (182). After phosphorylation of Thr197, the interaction cascade that is initiated leads to optimal orientation of Asp184 in the DFG motif for coordination of the γ-phosphate of ATP for phosphoryl transfer. It is believed that Mg1 interacts with the β- and γ-phosphates of ATP and Asp184, while Mg2 is bound by the α- and γ-phosphates of ATP and the side chain of Asn171 of the catalytic loop (179, 180).

We and others have shown the crucial importance of the Gly186 residue in Cα (Cα1 numbering) for catalytic activity. We performed a thorough search for naturally occurring mutations in the human *PRKACA* gene using both publicly available databases as well as through sequencing of exons 2–10 in 498 individuals (183). The search revealed several missense mutations, including Arg45Gln, Ser109Pro, Gly186Val, and Ser263Cys. Kinase activity and R subunit binding of the mutated C subunits was determined. Mutation of residues 45 and 263 did not significantly alter catalytic activity or R subunit binding. Mutation of Ser109 on the other hand led to decreased kinase activity, whereas R subunit binding was unaltered. Mutation of Gly186 to Val however, rendered the kinase completely inactive, and the resulting C subunit was unable to form holoenzymes with RI subunits, both confirming Gly186 as crucial for catalytic activity and instrumental in divalent cation binding.

**Figure 4:**
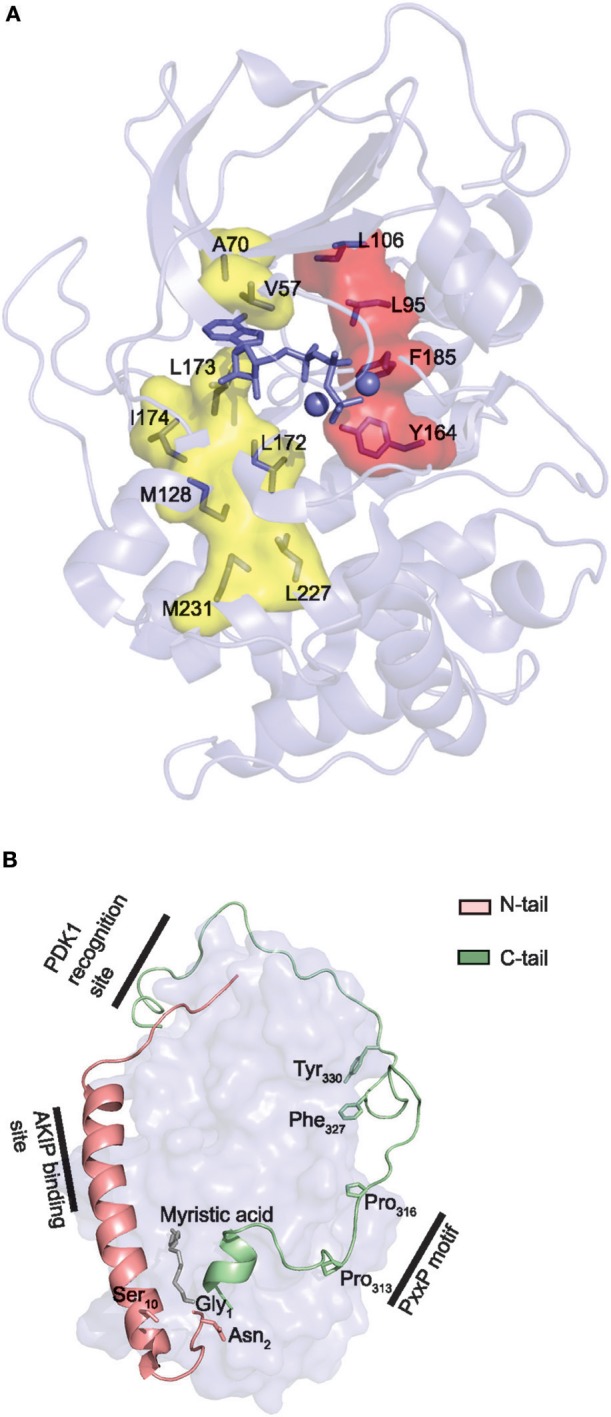
Core and tail structures of the PKA C subunit. **(A)** C- and R-spines in Cα1. In the active conformation of kinases, the C- and R-spines are assembled. In the case of PKA Cα1, the residues constituting the C-spine (yellow) are Ala70, Val57, Leu173, Ile174, Leu172, Met128, Met231, and Leu227. The adenine nucleobase of ATP (slate stick presentation) is also part of the C-spine. The R-spine (red) consists of Cα1 residues Leu106, Leu95, Phe185, and Tyr164. One-letter aa abbreviations are used in the figure. Figure is based upon (175). PDB identifier 3FJQ (169). **(B)** Presentation of the conserved kinase core (rendered as a surface in slate) of Cα1, including the N-tail (salmon) and C-tail (green) in cartoon presentations. Myristic acid (gray) is shown bound to the hydrophobic pocket. Selected structures and residues in the N- and C-tails are highlighted and described in the text. PDB identifier 1CMK (73).

The Local Spatial Pattern (LSP) alignment method, developed by Kornev et al. (175, 184), has revealed two conserved spatial motifs in ePKs. The motifs, termed Catalytic (C-) and Regulatory (R-) spines (Figure 4A), were identified as necessary structural motifs in catalytically active kinases. For an intact C-spine, the adenine nucleobase of ATP needs to bind to the active site. In PKA, the R-spine depends on phosphorylation of Thr197 in the activation loop. Equivalent Thr residues serve the same regulatory function in other kinases. Thus, assessment of the C- and R-spines in a kinase structure is helpful in determining whether a kinase is catalytically active or not.

The N- and C-terminal sequences outside of the kinase core are designated N- and C-tail, respectively (Figure 4B). Both tails are anchored to the core and facilitate its active conformation (85, 171). Except for PKI, C subunit interactions are predominantly mediated through binding to either the C- or N-tail (63, 75, 185). The C-tail is divided into three segments; the C-lobe tether (CLT), N-lobe tether (NLT), and the active site tether (AST). When PKA C is phosphorylated in the activation loop, the CLT and NLT are quite stable and seem to play an important allosteric role in organizing the active conformation (85). Most AGC kinases appear to be activated by the kinase PDK1, which is an AGC kinase itself, docking to the NLT of the C-tail (Figure 4B, “PDK1 recognition site”). Thus, the C-tail is a conserved structure in all AGC kinases (85). The CLT contains a PxxP (Pro-x-x-Pro) motif (residues 313–316 in PKA Cα1) (Figure 4B, “PxxP motif”), which is also conserved in almost all AGC kinases. The PxxP motif may represent a site for allosteric regulation of AGC kinase activity, possibly by binding to SH3 domains, which recognize Pro-rich regions (85, 186). The AST is more dynamic and contains Phe327 which is a part of the ATP binding site, and Tyr330 which is essential for the closed conformation of the enzyme (85).

## Structural relationships of the N-terminal end of the C subunit as determinates for three dimensional structure and subcellular targeting

There are isoform-specific sequence variations in the N-tail of the PKA C subunit. The role and function of Cα1 and Cβ1 N-terminal modifications are largely unknown but may influence localization, activity and interactions of the C subunit with other proteins (187).

Recently we performed alignments of exon 1-1 encoded Cα1/Cβ1 across species after the gene duplication yielding Cα and Cβ isoforms, and pre-duplication catalytic subunit [designated C1, (188)] residues reveals several conserved features in the N-terminus during evolution of the C subunit. Out of all 15 positions, position 1 and 2 is invariably Gly1 and Asn2 in both Cα1 and Cβ1, as well as C1, an ancient form of Cα and Cβ. In fact, Gly1 and Asn2 have been identified even in the C1 homologs of the early branching metazoans *Hydra vulgaris* (sequence identifier XP_002163934.1) and *Amphimedon queenslandica* (sequence identifier XP_011405630.1). It is well-established that Gly1 may have a function in mammals through its modification of myristoylation by the enzyme NMT (74). We have suggested that N-myristoylation of PKA C subunits is a feature found in all vertebrate Cα1/Cβ1 subunits, and maybe even all metazoan C1 subunits in general (188). N-terminal myristoylation of proteins appears to be an ancient mechanism, with the common ancestor gene of NMT possibly arising in early eukaryotic cells (189).

Deamidation of the neutrally charged residue Asn2 is likely the only way of obtaining a negatively charged residue adjacent to myristoylated Gly1, since the presence of a negatively charged residue next to Gly1 inhibits N-myristoylation (83, 190). Thus, the conservation of Asn2 may serve as a means for achieving myristoylated Gly1 and also obtaining a negatively charged aa number 2 through later deamidation of Asn2 to Asp2. Negatively charged residues next to myristoylated residues may contribute to promote their association to membranes. Studies from our group support the proposed cooperative function of Gly1 and Asn2, being both highly conserved. The β-Aspartyl shift mechanism (83), i.e., deamidation of Asn into Asp, occurs especially in the case of Asn being followed by residues Gly, Ser, or Ala (82). Indeed, alignment reveals that position 3 of vertebrate Cα1/Cβ1 subunits is either Ser, Ala, or Thr (188). Whether the rate of deamidation of Asn2 of isoforms such as Cβ1 of mouse, containing Thr3, is reduced (and as a consequence reduced phosphorylation of Ser10) has not been determined.

When exclusively studying vertebrate exon 1-1 encoded sequences except for lamprey, i.e., sequences after the C subunit gene duplication, six positions have been shown to be invariant (188). These are Gly1, Asn2, Lys8, Gly9, Glu11, and Ser14. Also, there is a high degree of conservation of similar aa properties among several of the positions that do vary among vertebrate Cα1/Cβ1 sequences. For example, residue 7 is either Lys or Arg which are both basic aa's, and the residue at position 13 is always occupied by an acidic aa, either Glu or Asp. The function of some of these residues has already been proposed. N-myristoylated proteins depend on an additional signal in order to associate with membranes. The basic residues in PKA Cα1/Cβ1 position 7 and 8 may serve this function, through forming electrostatic interactions with acidic phospholipids in membranes (191). Moreover, this is in line with the general N-terminal myristoylation motif, (M)GNXXXXRR (189, 192). Ser14 is solvent exposed, and potential functions of this residue are not known. Glu11 faces into the molecule and forms a salt bridge with Lys292, and may therefore contribute to structural stability. Hence it is reasonable to propose that Gly9 is structurally important in order for the N-terminus to adopt the right conformation, i.e., through the absence of potentially destructive steric hindrances.

Moreover, Ser10 is not highly conserved among vertebrate Cα1/Cβ1 homologs, despite its proposed role in regulation of myr-in/myr-out conformation (74). Aa position 10 has only been found to be Ser in mammalian Cα1 and Cβ1, in addition to selected amphibian and non-teleost fish Cα1 sequences. A more in depth analysis of different eutherian, i.e., placental mammalian, Cα1 and Cβ1 sequences from a range of species have revealed that Ser10 is conserved in all eutherian nucleotide sequences investigated. This may be interpreted as a possible case of convergent evolution of Cα1 and Cβ1 proteins in and it may be speculated that Ser10 has an important function in Cα1 and Cβ1, possibly unique to mammals, and may involve the phosphorylation switch. Moreover, the fact that Ser10 is pointing into solution, it is therefore likely that it is highly solvent exposed.

To be noted all the vertebrate species where the *PRKACA*/*PRKACB* exon 1-1 do not encode Ser10 the residue at position 10 is always Gly in Cα and Asn10 in Cβ (188). Due to the size and charge of Asn compared to Gly, this may further imply a larger fraction of Cβ in the myr-out conformation compared to Cα. This may further imply that mammalian C subunits have acquired a post-translational method through evolution of regulating myr-conformation (through phosphorylation/dephosphorylation of Ser10). This also suggests that most other vertebrates only regulate this feature at the level of transcription through transcribing either *PRKACA* or *PRKACB*.

**Figure 5:**
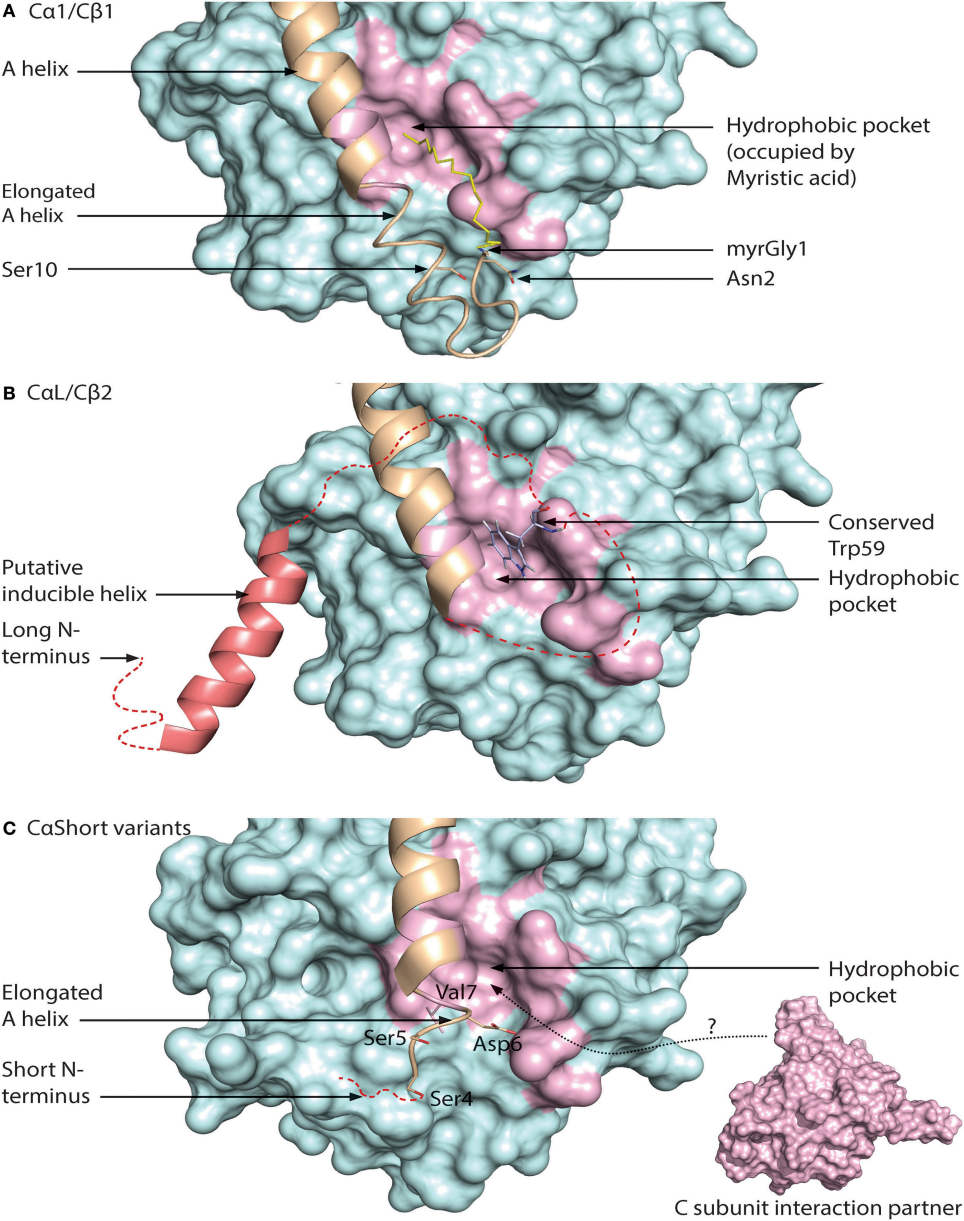
Different configurations of N terminal parts of C subunit isoforms. For all figures, the C subunit is represented in cyan with the hydrophobic pocket highlighted in purple. Alternative exon 1 encoded parts of the C subunit are in orange cartoon presentation, in addition to the mainly exon 2 encoded A helix. Hypothesized (i.e., not supported by published crystal structure data) structures of N-terminal residues are colored red. **(A)** Representation of human myristoylated Cα1. The structure of unphosphorylated (Ser10), unmodified (i.e., not deamidated) Asn2, and Gly1-myristoylated Cα1 shows a fully ordered N-terminus. The modifiable residues in the 5′ encoded exon are highlighted in stick presentations, and myristic acid (yellow) occupies the hydrophobic pocket. Based on the structure with PDB identifier 1CMK (73). **(B)** Proposed model of N-terminal structure of CαL/Cβ2 homologs. Our study identified a conserved Trp59 (human Cβ2 numbering) (stick presentation, slate) residue which potentially occupies the hydrophobic pocket. The most conserved part of the N-terminus was predicted to encode a helix structure, which we hypothesize may be ordered upon binding to interaction partners. The figure is modeled from the experimental structure of Cα1, with the N-terminal residues encoded by exon 1 modeled. PDB identifier 1CMK (73). **(C)** Proposed model of N-terminal structure of CαShort variants. Short N-terminal transcripts in Cα were identified in most vertebrate species investigated. The short N-terminal end displays the open hydrophobic pocket as earlier proposed for Cα2. The figure is based upon the experimental structure of human Cα2 with PDB identifier 4AE9 (63).

Recently, we also identified two positions that are consistently different in the mammalian Cα1 vs. Cβ1 N-terminus. Residue 5 is invariably Ala5 in Cα1 and Thr5 or Ile5 in Cβ1, whereas residue 12 is invariably Gln12 in Cα1 and Val12 in Cβ1. Gln12 is a more bulky and polar residue as opposed to the smaller and nonpolar aa Val12. Gln12 of Cα1 myr-in is oriented into the molecule, possibly stabilizing the N-terminus. Thus, it may be suggested that Val12 of Cβ1 makes the N-terminus more flexible and encourage the myr-out conformation. Alternatively, aa position 12 may serve other functions in the myr-out conformation, when it is solvent exposed and able to interact with other compounds. A model of N-terminal features associated with the Cα1 and Cβ1 proteins is shown in Figure 5A.

Exon 1-2 in *PRKACB* encodes 62 unique residues at the N-terminal end of Cβ2. Whereas Cβ2 is identified in all major vertebrate groups, a paralogous *PRKACA* exon 1-L encoding a long N-terminal Cα variant designated CαL has been identified at the mRNA level in all major vertebrate groups except for a likely loss in birds and mammals (188). The CαL/Cβ2 N-termini share several conserved features, by the fact that they firstly are all considerably longer than any other 5′ encoded exon variants of *PRKACA* and *PRKACB* identified. Secondly, the entire *PRKACA*/*PRKACB* exon 1-L/1-2 encoded N-termini have been predicted to be intrinsically disordered regions (IDRs). The identification of the CαL/Cβ2 N-termini as putative IDRs does not exclude the possibility that they are containing regions that transiently become ordered and bind to other macromolecules. Wiemann et al. predicted the presence of an amphipathic helix in the N-terminus of Cβ2 (67), and this putative α-helix encoding region was the most conserved part of the CαL/Cβ2 N-terminus when the two sequences were aligned. In fact, using DISOPRED3 prediction (193) it may be suggested that this part may be protein binding. We propose that the putative IDRs of the CαL/Cβ2 N-termini contain an inducible amphipathic α-helix which is formed when interacting with so far unidentified CαL-/Cβ2-specific interaction partners (Figure 5B, “Putative inducible helix”). Flanking the predicted α-helix are stretches of residues with less evolutionary pressure that are also predicted to be disordered. The segment spanning from the predicted α-helix to the start of peptide sequence encoded by exon 2 may function as a flexible linker, analogous to a fishing line with the inducible α-helix functioning as the “bait.” In fact, putative, long (>30 residues) disordered segments occur in more than 30% of eukaryotic proteins, and these proteins with IDRs are often involved in regulation of transcription and cell signaling (194). Coupled folding and binding provides a means for interactions of high specificity and relatively low affinity, which may be beneficial in signal transduction pathways, with the demand for transient signals and dissociation of proteins after a certain time (195).

Lacking the N-terminal Gly for myristoylation, we expect that the hydrophobic pocket of CαL/Cβ2 is either empty or occupied by structures other than myristic acid. In Cβ2 we identified a conserved Trp59 (human Cβ2 numbering) predicted to be located in proximity to the hydrophobic pocket (188). We speculate that Trp59 may function to occupy the entrance to the hydrophobic pocket, analogous to a lid, illustrated in Figure 5B (“Conserved Trp59”).

We have also identified short exons, which were called exon 1-S, in *PRKACA* of all major vertebrate groups except the Coelacanth (188). In mammals, including marsupials and monotremes, this was demonstrated to be orthologs of the previously identified human sperm-specific Ca2 protein exon 1-2. Later we found that the sperm-specific expression pattern was also conserved in all mammals which suggests that Cα2 serves a role for male fertility not only in mice (65), but possibly in mammals in general. Whether or not exons 1-S of non-mammalian species are orthologs of mammalian 1-S (i.e., exon 1-2) was not possible to be verified, as described in Søberg et al. (188). Alignments of the Cα2 proteins show low degree of conservation, and no obvious hydrophobic pocket-occupying properties. Purified human Cα2 protein has been demonstrated to be able to bind hydrophobic moieties (63). We therefore suggest that Cα2, and possibly CαS in general, is conserved through its ability to associate with other proteins or structures binding to the hydrophobic pocket (Figure 5C).

## Conserved heterogeneity in PKA C subunits suggests isoform-specific signaling pools

**Figure 6:**
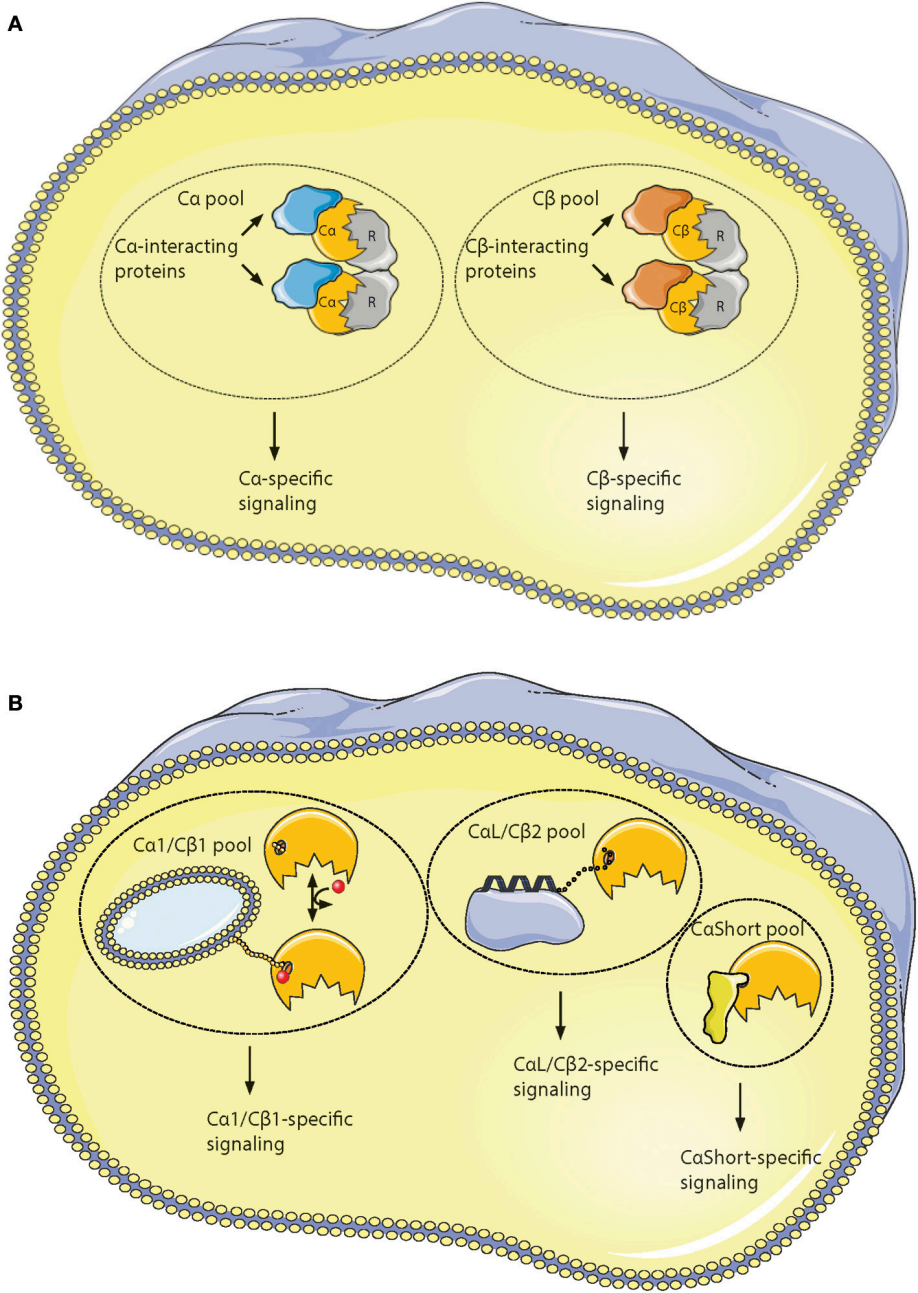
Hypothesis of localized pools of isoform-specific PKA signaling. **(A)** Most of the variations in the Core_16−350_ residues in Cα and Cβ proteins are located to 11 solvent exposed residues in the small lobe. This opens for the possibility of Cα- and Cβ-specific interaction partners interacting with the small lobe [described in (196)], possibly locating the two subunits into separate intracellular signaling pools. **(B)** Evolution of alternative N-termini in Cα and Cβ provides another mechanism for acquiring localized pools of isoform-specific PKA signaling [described in (188)]. The Cα1 and Cβ1 pool (left) shares the myristic acid with a regulatory mechanism, evolved in mammals, through phosphorylation/dephosphorylation of Ser10 for switching myristic acid in and out of the hydrophobic pocket (phosphate group presented as a red dot, and myristic acid presented as a yellow chain). This represents the main source of PKA C activity in most human cells. The conserved, putative inducible α-helix opens for the possibility of a CαL/Cβ2-specific pool (middle), docking the C subunits via a flexible linker to a CαL/Cβ2-specific subcellular assembly of proteins (purple). The Cα2 protein (CaShort pool, right) has a conserved sperm-specific expression in all mammals, and possibly interacts with Cα2-specific proteins (bright yellow) binding to the hydrophobic pocket. Similar CαShort-specific proteins may exist in other tissues in non-mammals. Figure created using the Servier Medical Art resource (http://www.servier.com/Powerpoint-image-bank).

It has been shown that all active Cα and Cβ splice variants are invariant in the core region encoded by *PRKACA* and *PRKACB* exons 2–10, respectively (196). This spans the residues 16–350 in Cα1. However, residues distinguishing Cα from Cβ are located to solvent-accessible loops in the small lobe as a signature motif distinguishing Cα from Cβ as paralogs. This together with the identification of key characteristics of the variable N-terminal Cα and Cβ splice variants has made us suggest the existence of PKA isoform-specific signaling pools. We propose that Cα and Cβ proteins may associate with Cα- and Cβ-specific C-KAPs, resulting in Cα- and Cβ-specific downstream signaling (Figure 6A). Similarly, splice variant-specific pools may be achieved through alternative interaction partners binding to the heterogeneous N-termini (Figure 6B).

**Figure 7:**
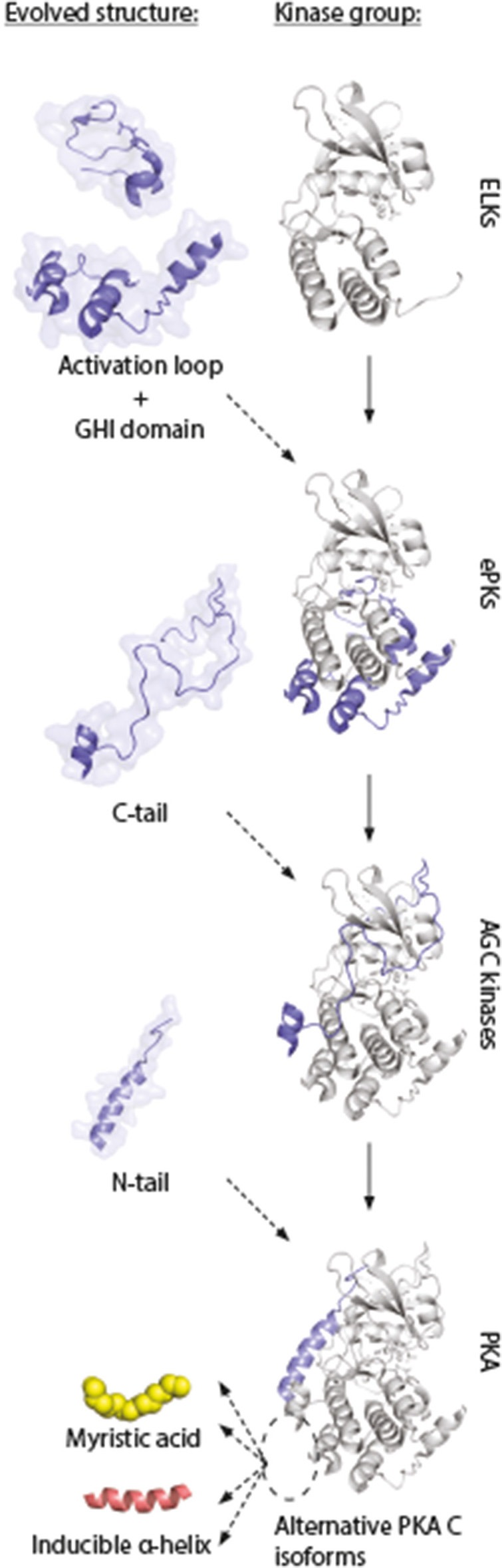
Model of evolution of PKA C subunits. The catalytic core is a conserved feature of the eukaryotic-like kinases (ELKs). The ePKs differ from ELKs through the attainment of the activation loop, typically involving a phosphorylatable Thr which can regulate the catalytic core into active/inactive conformations, and the G, H, and I helices (“GHI domain”), serving as docking motifs for substrates (197). The C-tail is a conserved feature of the AGC group of ePKs, and is highly regulated and essential for catalytic activity (171). The N-tail of PKA Cα and Cβ includes the A helix, which interacts with AKIP in Cα1 residues 15–29 (128). This segment is shared among all C subunit isoforms, whereas the alternative N-termini are located N-terminal to the AKIP-docking site. These alterations give rise to possible functional effects in different C subunit isoforms (“myristic acid,” “inducible helix,” “…”). Figure inspired by Taylor et al. (171, 197). PDB, 3FJQ.

This model supports the view of increased complexity and evolution into highly dynamic molecules in ePKs, where the conserved catalytic core contains most of the catalytic machinery necessary for enzymatic activity, whereas structures such as the N- and C-tails are involved in “fine-tuning” of PKA signaling through localization (197). This model also gives an understanding for why inactivation of kinase activity occurs when Gly186 is changed for a Val in Cα. This mutation is located in the highly conserved region of the kinase, shared among most PKs. The structures that are shared among all these kinases are typically important for catalysis. The evolved structures that build upon this framework (Figure 7, “activation loop,” “GHI domain,” “C-tail,” and “N-tail”) can be viewed as sophisticated modifications of PK structures, enabling a highly regulated PK, both in terms of kinase activity and protein/membrane interactions. We suggest that the alternative PKA C isoforms represent an extension of this concept, and show the conservation of such alternative modifications located to the area around the hydrophobic pocket (Figure 7, dashed ellipse) and the small lobe. Similar increases in complexity in PKA signaling has been reported in other parts of the PKA signaling pathway, such as increases in R subunit and AKAP encoding genes throughout evolution (198, 199).

## Are off-target effects of PKA C subunit associated with disease?

Evidence for the idea that targeting PKA C subunit activity in space and time is crucial for regulating PKA catalytic activity has emerged over the past decades. The first evidence for this was demonstrated in mice by Cummings and coworkers who showed that RIα compensation in RIIβ null mutated was associated with dislocated PKA holoenzyme and increased basal PKA C subunit activity not regulated by cAMP due to lower affinity for the C subunit by RIα compared to RIIβ (51). Additional support for this idea came from studies on RIα ablation in mice which is associated with severe developmental defects. Interestingly, the phenotype could be rescued by crossing the RIα ablated mice with the Cα null mutated mice, suggesting that the phenotype is caused by abrogated regulation of C subunit activity (200). In man, the same research group also showed that haploinsufficiency at the *PRKAR1A* locus found in ~75% of patients suffering from Carney complex (CNC), in addition to suffer from spotty skin pigmentation, cardiac and cutaneous myxomas, and endocrine tumors such as micronodular adrenocortical hyperplasia (MAH), these proteins also had reduced fertility due to unregulated C subunit activity in male germ cells (201). Interestingly, Forlino and coworkers reported that a young woman (19 of age) who suffered from CNC and who had developed MAH carried a triplication of chromosome 1p31.1. Chromosome 1p31.1contains the *PRKACB* gene which led the authors of this paper to suggest that this patient encoded increased levels of Cβ. Based on this they further suggested that increased C subunit activity may be associated with disease pathogenesis and development of MAH (202).

Increased C subunit activity associated with disease pathogenesis is further supported by the identification of a frequent mutation in the *PRKACA* gene. The mutation Leu205Arg is located in the P+1 loop of Cα1 but not Cβ1, rendering the C subunits insensitive to inhibition by the R subunits (203). This hotspot mutation was demonstrated to be the likely cause of nearly 70% of cortisol-producing adrenocortical adenomas (C-PAA) in Cushing patients (204). This finding was verified in a study by others (205).

Finally, two recent studies hamper the importance of locating C subunit activity. In fibrolamellar hepatocellular carcinoma (FL-HCC), which is a rare liver tumor, a ~400-kilobase deletion on chromosome 19 leads to a chimeric gene product consisting of exon 1, also including parts of exon 2, of *DNAJB1* fused with exon 2–10 of *PRKACA* (*DNAJB1-PRKACA*) (206, 207). The resulting DNAJB1-Cα1 fusion protein retains R subunit binding capacity, and basal PKA C kinase activity in FL-HCC cell lysates is comparable that of lysates from normal liver cells. However, DNAJB1-Cα has a reduced avidity for R subunits, and catalytic activity is greatly increased upon cAMP stimulation compared to WT Cα (29). In addition, the fusion transcript is expressed 10-fold higher than *PRKACA* (29). Moreover, the fusion protein interacts with β-catenin, and overexpression of WT Cα does not fully recapitulate the oncogenic activity of DNAJB1-Cα1. The latter may suggest that the pathogenesis of FL-HCC is not only dependent on altered PKA C activity but also on localization (208). Taken together these reports provide evidence that gain of function of Cα and Cβ, respectively, provide two non-identical phenotypes (MAH and C-PAA) suggesting non-redundant functions. In addition, dislocation of PKA C subunits leads to off-target effects which are associated with disease.

## Author contributions

KS has in collaboration with BS written and carefully revised the draft for this review. KS has in collaboration made the drafts and final layout of the figures.

### Conflict of interest statement

The authors declare that the research was conducted in the absence of any commercial or financial relationships that could be construed as a potential conflict of interest.
